# The Work of the Holy Ghost

**Published:** 1887-05-21

**Authors:** 


### Words of Consolation.

[Communications for this column should be addressed, "Chaplain," care of the Editor of The Hospital, who will gratefully welcome any suggestions or inquiries from matrons, nurses, and the staff generally.]

XXXIV.—The Work of the Holy Ghost.

We can some of us call to mind general convictions we have had in times past of our lives being wrong, and yet we knew not how wrong, or how grievously we had sinned, until the yet deeper sense of sin was given. "Against Thee only have I sinned and done this evil in Thy sight" (Ps. li, 4). "Father, I have sinned against heaven and before Thee (Luke xv, 18). Then we may have felt inclined to despair at this sight of our own hearts, revealed at last in the true light, not feeling at the very time that this was all the working of the Holy Ghost. But it was so. Moreover, this deepening of conviction, with its accompanying sorrow, must be allowed to proceed yet further with many of us, or we shall never fully realise our need of a Saviour, not only to atone for sin, but who must also cleanse us from its guilt and power.

Consider another truth which should deepen conviction. It is on account of the special indwelling of the Holy Ghost that sin committed wilfully by a Christian has a much deeper dye than any other committed, say, by a heathen, or by those who have been brought up in ignorance of the Baptismal Covenant. All sin against God is evil. But besides this, sin in the baptised has the nature of sacrilege. "Know ye not that ycur body is the temple of the Holy Ghost, which is in you?" (1 Cor. vi, 18). Therefore, sin in us, especially impurity, is not merely against a God universally present, but is in addition, as is were, the desecration of a temple which has been set apart as the shrine of His presence. The closeness of the Divine presence increases the sinfulness of sin. We must therefore estimate all our faults by the standard of the New Testament. Again, if we accept this high standard, we shall find that the same Spirit who has told us so much of the sinful past, and given us a good hope of righteousness in Christ, will keep on telling us of the judgment to come. Hearts alive to His convictions, and submissive to His teaching, are ever filling too with the spirit of holy fear, dreading sin because they believe in the strict account they will have to render, clinging to Christ yet more closely, because they would make Him more entirely theirs as the Saviour before He comes to be their Judge.

You can call to mind, perhaps, how thoughts of the judgment have come into your mind when you scarce expected them. You could not resist them altogether. The day has been pictured to your imagination, and you fancied yourself before the great white throne, awaiting the sentence for weal or woe. That conviction has also come from the Holy Ghost. But He does noc terrify souls with pictures of the judgment before He has given them a knowledge of the love of Christ, and shown them how they may be clothed in the fine linen which is the righteousness of the Saints. God grant that our convictions may lead to such a clothing in righteousness while we are here, to such perseverance in the spirit of God's holy fear, that we may look forward in confidence to "the day of His appearing."

(*To be continued.*)

